# INHBA gene silencing inhibits proliferation, migration, and invasion of osteosarcoma cells by repressing TGF-β signaling pathway activation

**DOI:** 10.1186/s13018-023-04330-2

**Published:** 2023-11-08

**Authors:** Hongyu Zhang, Yuemei Huang, Qiuting Wen, Yan Li, Lin Guo, Na Ge

**Affiliations:** 1 Second Department of Orthopaedics, The Third Affiliated Hospital of Qiqihar Medial University, Qiqihar, 161000 China; 2 Wuzhou Red Cross Hospital, Wuzhou, 543002 China; 3 Department of Clinical Pathology, College of Qiqihar Medical University, Qiqihar, 161006 China; 4 The First Hospital of Qiqihar, Qiqihar, 161005 China; 5 Department of Radiology, The Third Affiliated Hospital of Qiqihar Medial University, No. 27 Taishun Street, Qiqihar, 161000 China

**Keywords:** INHBA, Osteosarcoma, Cell proliferation, migration, and invasion, TGF-β signaling pathway

## Abstract

**Background:**

Osteosarcoma (OS) is a refractory malignancy. This study aimed to explore the roles and mechanisms of Inhibin subunit beta A (INHBA) in OS.

**Methods:**

INHBA expression levels in OS tissues and cells were assessed using RT-qPCR and western blotting. The impact of INHBA silencing on OS development was then explored by transfecting the OS cell lines U2OS and MG63 with INHBA-small interfering RNA (siRNA). The influence of INHBA silencing on U2OS and MG63 cell proliferation, migration, and invasion was examined using MTT and Transwell assays. Epithelial–mesenchymal transition (EMT) markers (E-cadherin and N-cadherin) were analyzed by RT-qPCR. The expression of genes involved in cell proliferation, migration, invasion, and the TGF-β signaling pathway was evaluated by western blotting and RT-qPCR.

**Results:**

INHBA levels were elevated in the OS tissues and cells. Furthermore, the transforming growth factor-β (TGF-β) signaling pathway of OS cells was suppressed in response to INHBA-siRNA, whereas proliferation, migration, and invasion of OS cells were inhibited. Besides, INHBA-siRNA significantly inhibited OS cell EMT, evidenced by enhanced E-cadherin mRNA expression and reduced N-cadherin mRNA expression. Further mechanistic studies revealed that the TGF-β1 agonist SRI-011381 hydrochloride increased OS cell proliferation, migration, and invasion after INHBA downregulation.

**Conclusion:**

We found that INHBA silencing could play a vital role in OS via TGF-β1-regulated proliferation, migration, and invasion.

## Introduction

Osteosarcoma (OS) is a high-incidence malignant bone cancer usually occurring in adolescents and older adults [1]. OS generally develops in mesenchymal cell lines and directly or indirectly forms osteoid tumor tissues at the cartilage stage [2]. Clinically, OS often presents with pain at the tumor site, associated with tumor tissue erosion and lysis of the bone cortex [3]. However, the pathogenesis of OS remains unclear, and there is no effective treatment. Current treatments include surgical resection, adjuvant chemotherapy, and multidrug neoadjuvant therapy [4]. Although treatment has improved, patient prognosis is poor, and survival rates remain low. Therefore, it is necessary to thoroughly investigate the pathogenesis of OS and develop effective treatment methods. This will help to identify new biomarkers and therapeutic targets for OS.

Studies have found that apoptosis and necrosis of tumor cells are important indicators for treating OS, and inhibiting apoptosis contributes to their proliferation and migration [5]. Transforming growth factor-β (TGF-β) regulates various cell biological processes, including cell proliferation and migration, and affects downstream Smad and non-Smad pathways [6–8]. The multifunctional cytokine TGF-β has long been recognized as an immune-suppressive factor in the tumor microenvironment [9]. Studies have reported that TGF-β has promoting effects on OS, and the level of TGF-β in the serum of patients with OS was significantly higher than normal [10, 11]. Furthermore, increased TGF-β levels are associated with tumorigenesis [12, 13]. Moreover, TGF-β has been reported to promote the malignant biological behaviors of OS cells [14, 15]. Therefore, suppressing the TGF-β signaling pathway could protect against OS development.

Inhibin subunit beta A (INHBA), a member of the TGF-β superfamily, is located at 7p14.1 [16, 17]. INHBA forms Activin A by homodimerization or Inhibin by heterodimerization [18]. INHBA plays an important role in the progression of various (including esophageal, breast, lung, and gastric) cancers [19–23]. In addition, it is upregulated in various tumors and participates in tumor proliferation and migration [24]. For example, INHBA knockdown suppresses the proliferation and invasion of nasopharyngeal carcinoma cells [25]. Silencing INHBA inhibits invasion and migration in gastric cancer through the TGF-β signaling pathway [23, 26]. INHBA is a prognostic predictor in patients with colon adenocarcinoma [27]. Recent studies have shown that INHBA is elevated in breast cancer tissues and promotes the metastasis of breast cancer cells [20, 21]. Study also revealed that INHBA plays a functional role in supporting EMT phenotype of breast cancer cells [20]. However, the mechanism of action of INHBA in OS remains unknown.

This study aimed to explore the expression of INHBA in patients with OS and to analyze the effect on the proliferation, migration, and invasion of OS cells.

## Materials and methods

### Clinical specimens

We selected 30 patients with OS who were treated at the Third Affiliated Hospital of Qiqihar Medical University. Their OS and adjacent normal tissues were obtained by surgical resection. OS was confirmed by tumor pathology and genetics and reconfirmed by two senior pathologists. Samples were stored at – 80 °C until RNA extraction. The basic characteristics of the patients are shown in Table 1. This study was approved by the Ethics Committee of the Third Affiliated Hospital of Qiqihar Medical University, and all participants provided written informed consent.

**Table 1:** The basic information of osteosarcoma patients

Characteristics		Cases (*n* = 30)
*Age (years)*		
< 25		8
≧ 25		22
*Gender*		
Male		15
Female		15
*Tumor size (cm)*		
< 8		18
≧ 8		12
*Tumor stage*		
I		8
II/III	22	
*Metastasis*		
Absent	9	
Present	21	

### Bone scan and SPECT-CT diagnosis [28]

Tc-hydroxymethyldiphosphate (Tc-HMDP) was administered intravenously. After the patient rested horizontally for 1–2 h, a flat bone scan was performed using a SPECT/CT scanner with a high-resolution collimator. The following parameters were used to obtain scanned images: voltage, 20 kV; tube current, 70–260 mA; and scan time, 10 s. During the scan, the SPECT machine was rotated at 1 s/turn, and these images were sent to a computer to acquire single-photon emission computed tomography images.

### Reverse transcription-quantitative polymerase chain reaction (RT-qPCR)

Total RNA was extracted from OS tissues (from patients) and OS cell lines MG63 and U2OS using RNA-easy Isolation Reagent (Vazyme Biotech Co., Nanjing, China). We obtained cDNA using reverse transcription kits (Vazyme Biotech Co.) according to the manufacturer’s instructions. RT-qPCR was subsequently performed with SYBR Green (Vazyme Biotech Co.). The primers were synthesized by Sangon Biotech (Shanghai, China) with the following sequences: INHBA forward 5'-CCTCCCAAAGGATGTACCCAA-3' and reverse 5'-CTCTATCTCCACATACCCGTTCT-3'; Proliferating Cell Nuclear Antigen (PCNA) forward 5'-CCTGCTGGGATATTAGCTCCA-3' and reverse 5'-CAGCGGTAGGTGTCGAAGC-3'; Cyclin D1 forward 5'-GCTGCGAAGTGGAAACCATC-3' and reverse 5'-CCTCCTTCTGCACACATTTGAA-3'; Matrix Metalloproteinase 9 (MMP9) forward 5'-TGTACCGCTATGGTTACACTCG-3' and reverse 5'-GGCAGGGACAGTTGCTTCT-3'; MMP-2 forward 5'-TACAGGATCATTGGCTACACACC-3' and reverse 5'-GGTCACATCGCTCCAGACT-3'; TGF-β1 forward 5'-GGCCAGATCCTGTCCAAGC -3' and reverse 5'-GTGGGT TTCCACCATTAGCAC-3'; Smad4 forward 5'-CTCATGTGATCTATGCCCGTC-3' and reverse 5'-AGGTGATACAACTCGTTCGTAGT-3'; Smad7 forward 5'-TTCCTCCGCTGAAACAGGG-3' and reverse 5'-CCTCCCAGTATGCCACCAC-3'; E-cadherin forward 5'-GACACTGGTGCCATTTCCAC-3' and reverse 5'-AGTTCGAGGTTCTGGTATGGG-3';

N-cadherin forward 5'-AGAGGCAGAGACTTGCGAAAC-3' and reverse 5'-ACACTGGCAAACCTTCACGC-3'; GAPDH forward 5'-GGAGCGAGATCCCTCCAAAAT-3' and reverse 5'-GGCTGTTGTCATACTTCTCATGG-3'. The relative mRNA expression was calculated as 2^ΔΔCt^.

### Western blotting

Cells were lysed with RIPA lysis buffer (Beyotime Biotechnology, Shanghai, China) containing protein inhibitors, and the supernatant was collected by centrifugation at 12,000 rpm to obtain the total protein. Total protein was measured with a BCA kit (Thermo Fisher Scientific,). Equal amounts of protein (20 µg) were separated by sodium dodecyl sulfate–polyacrylamide gel electrophoresis (SDS-PAGE) and transferred to polyvinylidene difluoride (PVDF) membranes (Millipore). The membranes were blocked with 5% bovine serum albumin (BSA). After 1 h, the membranes were incubated overnight at 4 °C with primary antibodies against INHBA (ab128958, 1: 1000; Abcam), PCNA (#13110, 1: 3000; CST), Cyclin D1 (26939–1-AP, 1: 2000; Wuhan Sanying Biotechnology), MMP-2 (ab181286, 1: 500; Abcam), MMP9 (ab76003, 1: 500; Abcam), TGF-β1 (21898–1-AP, 1: 1000; Wuhan Sanying Biotechnology), p-Samd2 (#18338, 1: 1000; CST), p-Smad3 (#9520, 1: 500; CST, Smad4 (PA5-34806, 1: 1000; Thermofishe), Smad7 (25840–1-AP, 1: 2000; Wuhan Sanying Biotechnology), and GAPDH (ab181602, 1: 10000; Abcam). The next day, the membranes were washed five times for 5 min each with TBST buffer. The membranes were then incubated with HRP-labeled secondary antibodies. After 2 h, the bands were visualized using ECL luminescent solution.

### Cell culture and transfection

Human osteoblastic cell line (hFOB1.19) and human osteosarcoma cell lines (U2OS and MG63) were obtained from the American Type Culture Collection (ATCC; Manassas, VA, USA). The cells were cultured in Dulbecco’s modified DMEM medium (Basal Media) containing 10% fetal bovine serum (FBS; Biological Industries). Cells were cultured in an incubator at 37 °C and 5% CO_2_.

INHBA-siRNA (sc-39783, Santa Cruz Biotechnology, Santa Cruz, CA, USA) and control-siRNA (sc-36869, Santa Cruz Biotechnology) were used to investigate the role of INHBA in OS. 0.2 μM INHBA-siRNA or 0.2 μM control-siRNA was transfected into U2OS or MG63 cells using Lipofectamine 3000 (Life Technologies) according to the manufacturer’s instructions.

### Drug treatment

To investigate the role of the TGF-β1 signaling pathway in the effect of INHBA on OS, we treated the transfected U2OS or MG63 cells with 10 μM SRI-011381 hydrochloride (TGF-β1 agonist). The treatment groups were as follows: control-siRNA, INHBA-siRNA, and INHBA-siRNA + SRI-011381.

### MTT assay for cell viability

Cell proliferation was measured using the MTT assay, according to a previous report [29]. Briefly, 2 × 10^3^ cells/well were seeded in 96-well plates. After 24 h, 20 µl MTT solution (Beyotime) was added to each well, and cells were cultured at 37 °C for 4 h. Absorbance levels were measured at 570 nm with a plate reading spectrophotometer. Data are expressed as mean ± SD.

### Transwell assay for migration and invasion

Cell migration and invasion were assessed using Transwell assays as previously described [30]. Briefly, 8 μm pore-size Transwell chambers were added to the upper and lower layers with serum-free and 10% serum-containing cultures, respectively, and the cells were placed in the upper layer for 24 h. Cells penetrating the lower layer were subsequently fixed using 4% methanol and stained with crystal violet. Cells were counted from five random fields using an inverted microscope (LEICA). The difference between cell migration and invasion experiments was whether the Transwell chambers were pre-coated with Matrigel (BD Biosciences, San Jose, CA, USA).

### Statistical analysis

Statistical analyses were performed using GraphPad Prism software, and differences between groups were analyzed using one-way analyses of variance (ANOVAs) or unpaired Student’s t tests. All data are presented as mean ± SD; *p* < 0.05 was considered statistically significant.

## Results

### Expression of INHBA in cancerous tissues of osteosarcoma patients

**Fig. 1:**
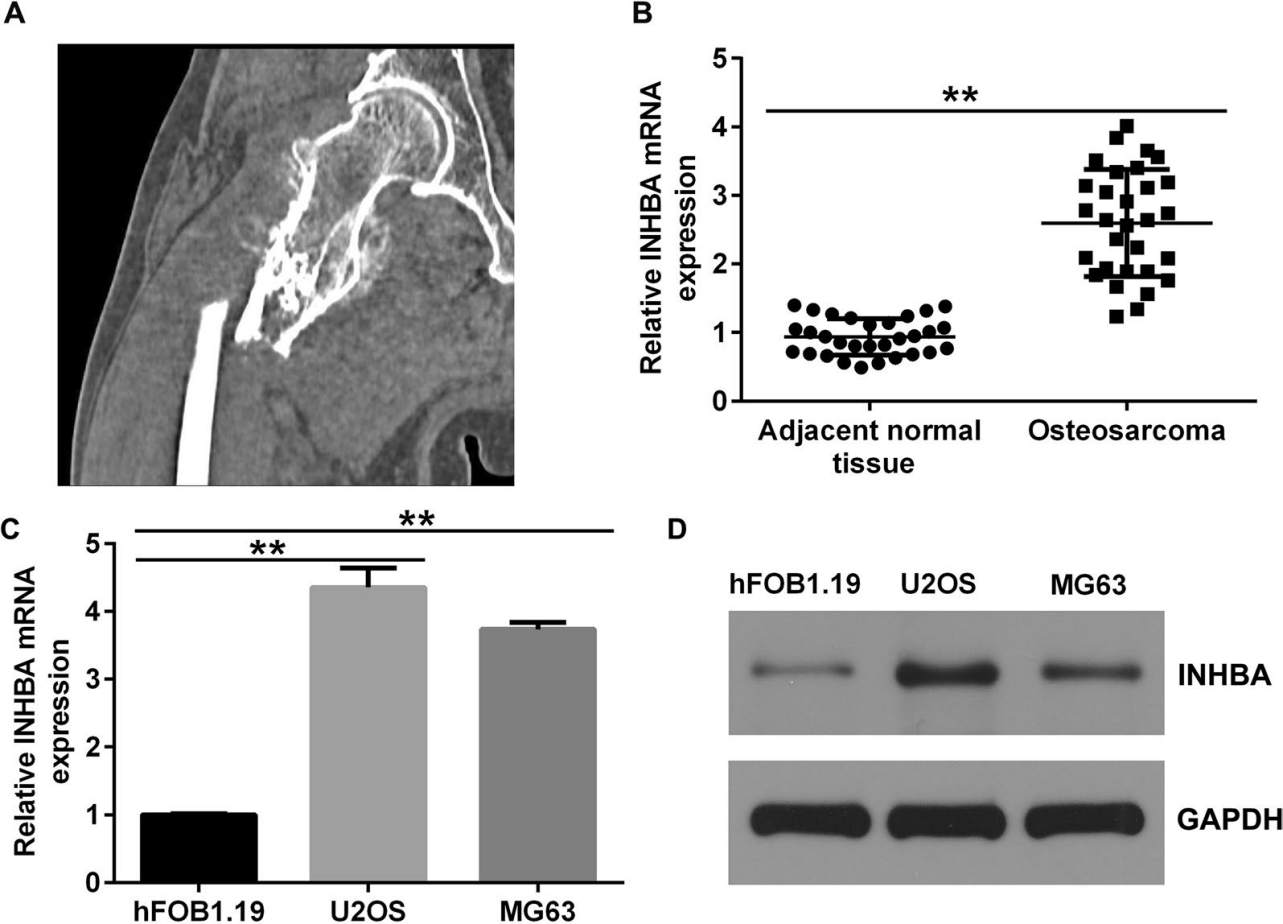
INHBA expression in cancer tissues from patients with OS and OS cell lines. **A** SPECT-CT diagnostic image of OS; **B** INHBA expression in the tissues of patients with OS using RT-qPCR. The mRNA and protein levels of INHBA in OS cell lines were detected by **C** RT-qPCR and **D** western blotting.**p < 0.01

To explore the role of INHBA in OS, we obtained 30 OS samples and paired them with adjacent normal tissue specimens from patients who underwent resection at the Third Affiliated Hospital of Qiqihar Medical University. All patients with OS were diagnosed using SPECT/CT (Fig. 1A), and two senior pathologists confirmed the diagnosis. Moreover, INHBA levels were detected by RT-qPCR. INHBA expression was enhanced in human OS samples compared to adjacent normal tissues (Fig. 1B), indicating that INHBA was upregulated in osteosarcoma cells.

### Expression of INHBA in osteosarcoma cells

Next, we examined INHBA levels in OS cell lines using western blotting and RT-qPCR. INHBA expression was significantly higher in human OS cell lines (U2OS and MG63) than in the normal osteoblast cell line (hFOB1.19, Fig. 1C, D).

### Silencing of INHBA regulates the proliferation, migration, and invasion of OS cells

**Fig. 2:**
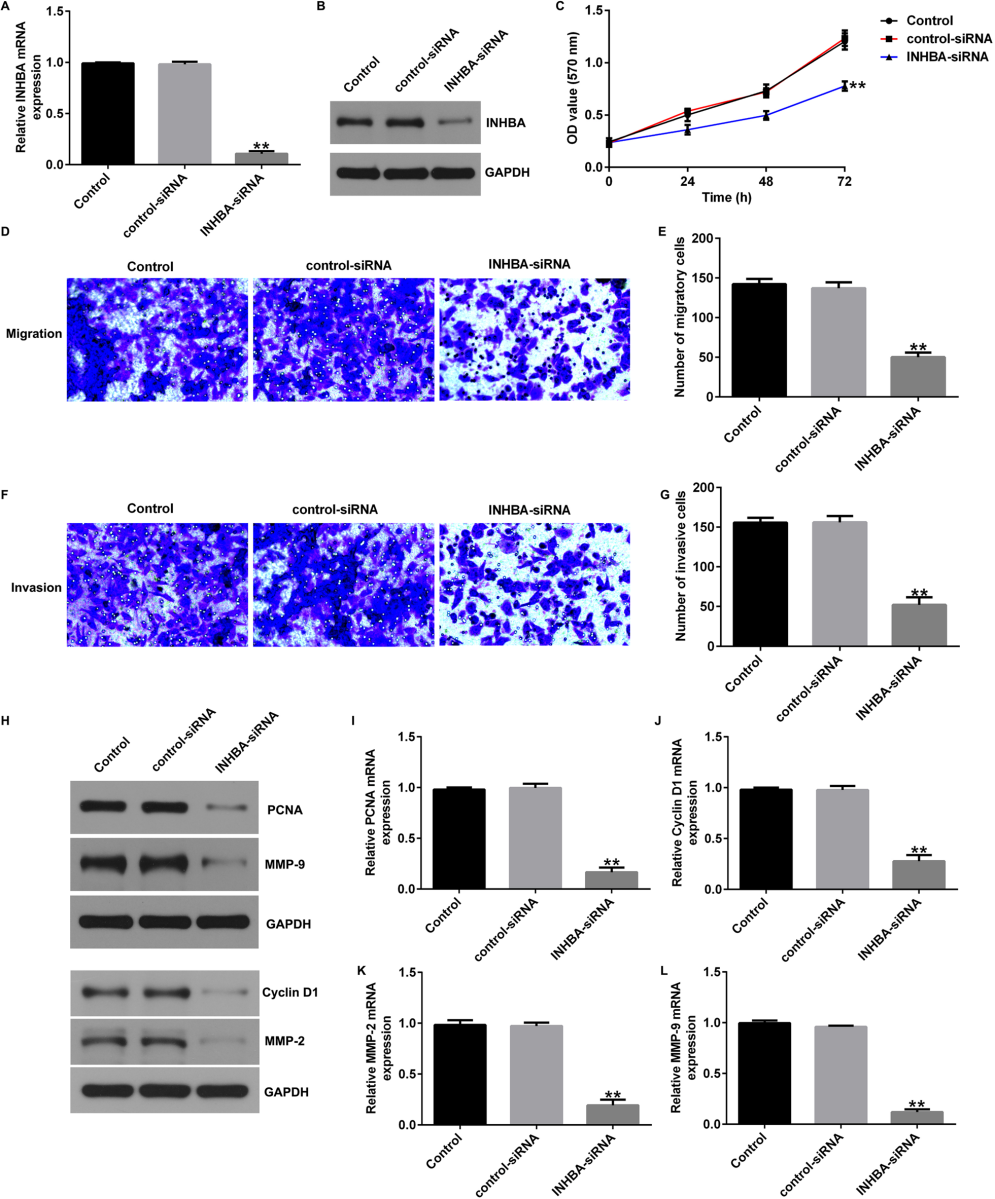
Effects of INHBA silencing on proliferation, migration, and invasion of U2OS cells. **A** RT-qPCR analysis of *INHBA* in U2OS cells transfected with control-siRNA or INHBA-siRNA; **B** Western blot analysis of INHBA in U2OS cells transfected with control-siRNA or INHBA-siRNA; **C** An MTT assay was used to evaluate the cell proliferation of U2OS cells; **D** and **E** A Transwell assay was used to evaluate the migration of U2OS cells; **F** and **G** A Transwell assay was used to evaluate the invasion of U2OS cells; **H**–**L** Analysis of PCNA, Cyclin D1, MMP-2, and MMP9 protein and mRNA expression in U2OS cells by western blotting and RT-qPCR. **p < 0.01 versus control-siRNA group

**Fig. 3:**
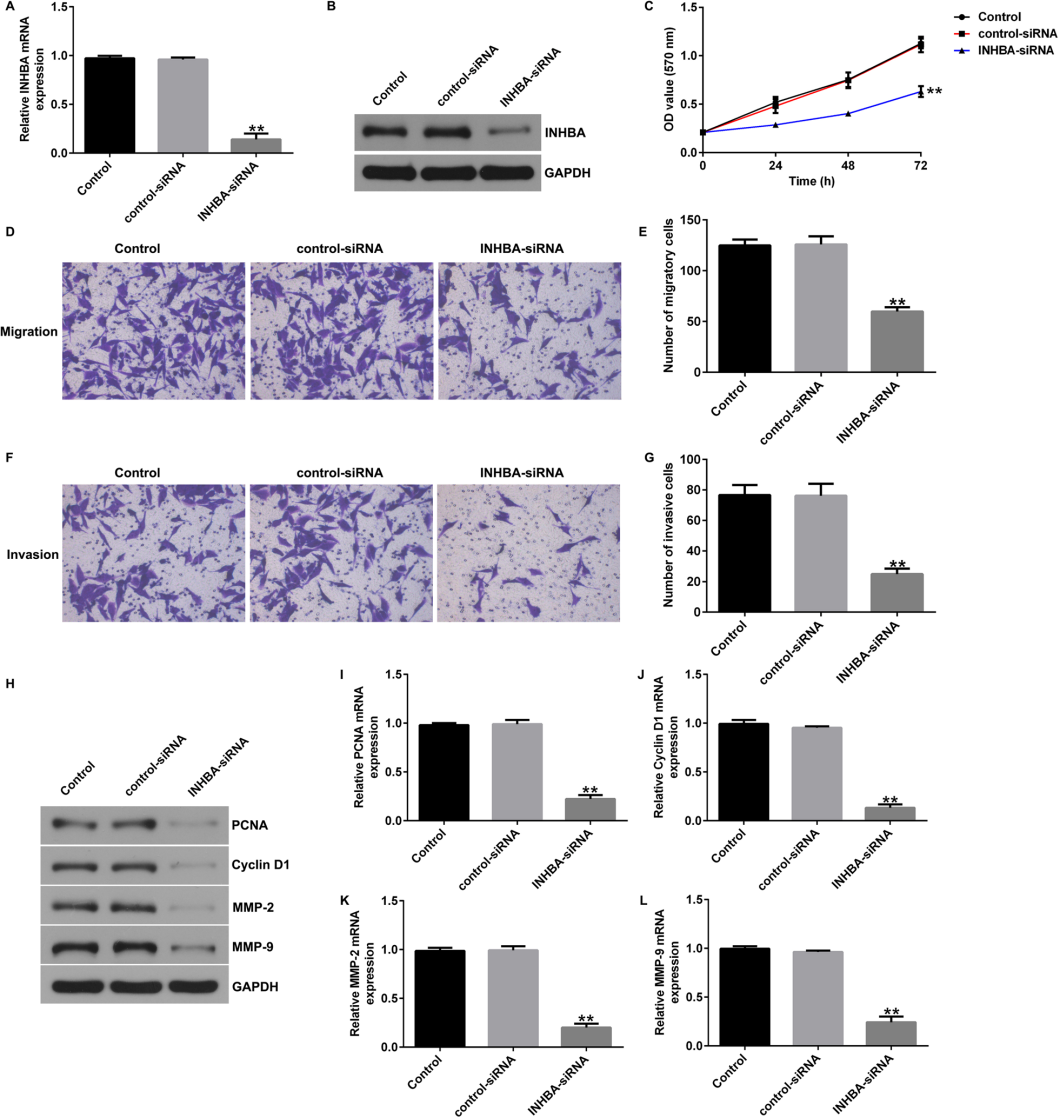
Effects of INHBA silencing on proliferation, migration, and invasion of MG63 cells. **A** RT-qPCR analysis of *INHBA* in MG63 cells transfected with control-siRNA or INHBA-siRNA; **B** Western blot analysis of INHBA in MG63 cells transfected with control-siRNA or INHBA-siRNA; **C** An MTT assay was used to evaluate the cell proliferation of MG63 cells; **D** and **E** A Transwell assay was used to evaluate the migration of MG63 cells; **F** and **G** A Transwell assay was used to evaluate the invasion of MG63 cells; **H**–**L** Analysis of PCNA, Cyclin D1, MMP-2, and MMP9 protein and mRNA expression in MG63 cells by western blotting and RT-qPCR. **p < 0.01 versus control-siRNA group

We further explored the role of INHBA in OS by establishing a stable INHBA-knockdown OS cell line. After transfection of control-siRNA and INHBA-siRNA into U2OS or MG63 cells, proliferation, migration, and invasion were examined using MTT and Transwell assays. INHBA expression in U2OS cells was significantly reduced by INHBA-siRNA (Fig. 2A, B). Moreover, INHBA-siRNA inhibited the proliferation (Fig. 2C), migration (Fig. 2D, E), and invasion (Fig. 2F, G) of U2OS cells. In addition, western blotting and RT-qPCR showed that INHBA downregulation significantly inhibited the protein and mRNA levels of PCNA, Cyclin D1, MMP-2, and MMP9 (Fig. 2H–L). Similar results were obtained in MG63 cells (Fig. 3). These results suggest that INHBA silencing inhibits OS cell proliferation, migration, and invasion.

### Silencing of INHBA blocks the TGF-β signaling pathway in OS cell lines

**Fig. 4:**
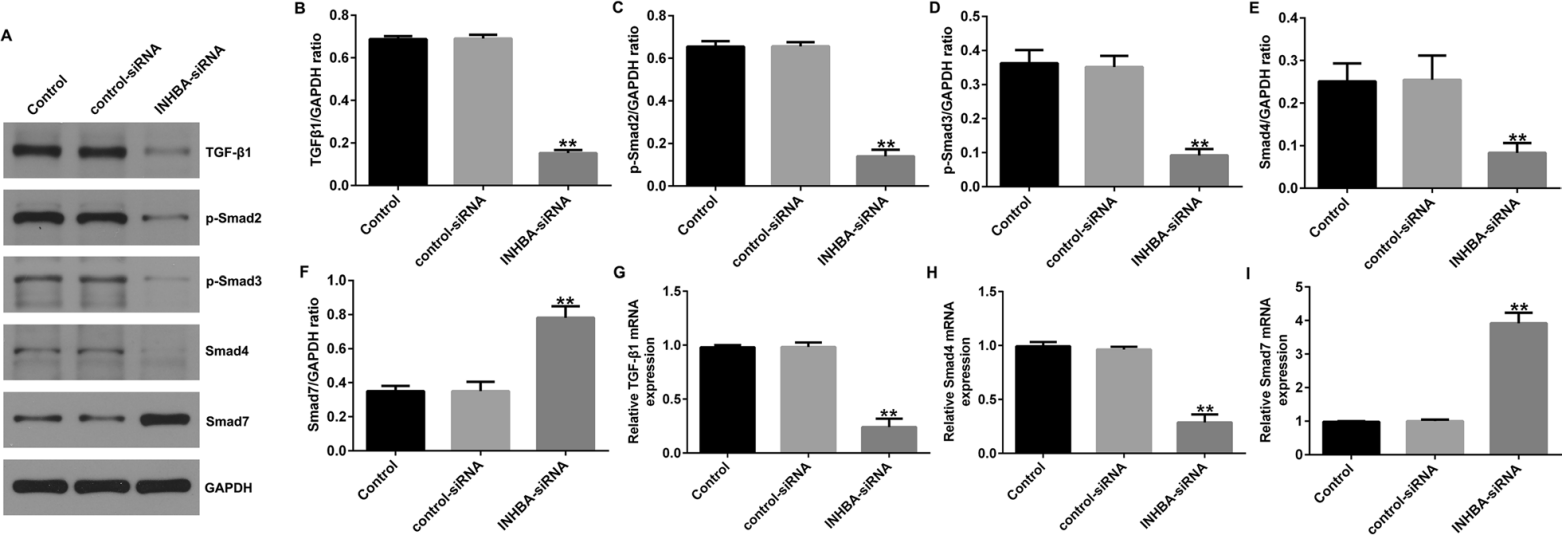
Effect of INHBA silencing on the TGF-β signaling pathway in U2OS cells. A-I. western blotting and RT-qPCR detected the protein and mRNA expression of genes related to the TGF-β signaling pathway in U2OS cells. **p < 0.01 versus control-siRNA group

**Fig. 5:**
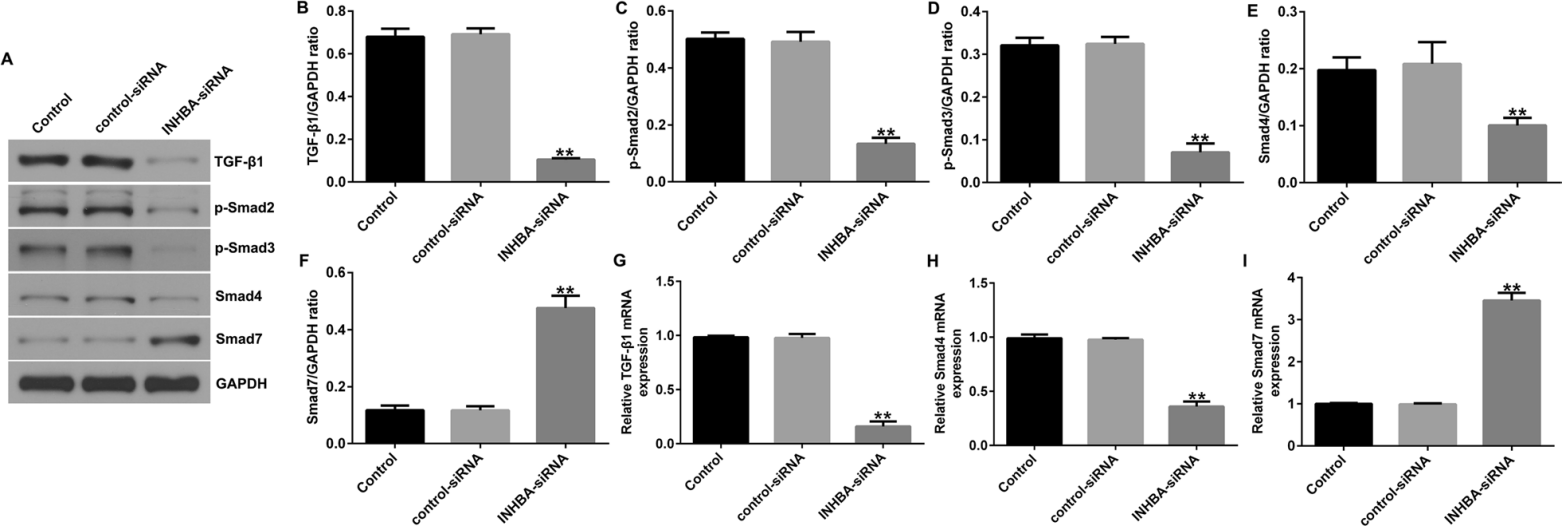
Effect of INHBA silencing on the TGF-β signaling pathway in MG63 cells. **A-I**. western blotting and RT-qPCR detected the protein and mRNA expression of genes related to the TGF-β signaling pathway in MG63 cells. **p < 0.01 versus control-siRNA group

Recent research has reported that INHBA participates in the migration and invasion of gastric cancer cells by regulating the TGF-β signaling pathway. Therefore, we speculated that INHBA silencing could regulate the proliferation, migration, and invasion of OS cells by inhibiting TGF-β signaling. We detected proteins of the TGF-β signaling pathway using western blot and RT-qPCR. Downregulation of INHBA significantly inhibited TGF-β1, p-Smad3, p-Smad2, and Smad4 levels and promoted the expression of Smad7 in U2OS cells (Fig. 4A–F) and in MG63 cells (Fig. 5A–F). In addition, compared to the control-siRNA group, INHBA-siRNA significantly inhibited the mRNA levels of Smad4 and TGF-β1 and promoted the mRNA level of Smad7 in U2OS cells (Fig. 4G–I) and in MG63 cells (Fig. 5G–I).

### Activation of the TGF-β signaling pathway reverses the effect of INHBA knockdown on OS cells

**Fig. 6:**
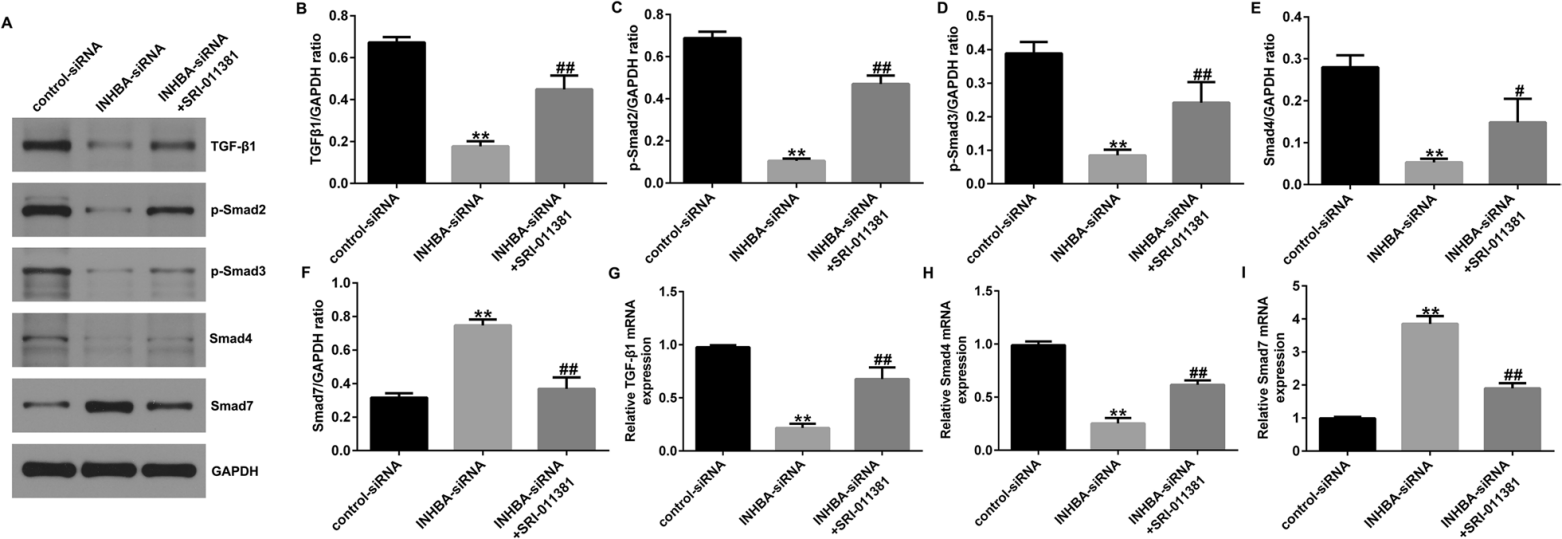
TGF-β1 agonist reversed the effects of INHBA silencing on the TGF-β signaling pathway in U2OS cells. **A**–**I** western blotting and RT-qPCR detected the protein and mRNA expression of genes related to the TGF-β signaling pathway in U2OS cells. **p < 0.01 versus control-siRNA group; #, ##*p* < 0.05, 0.01 versus INHBA-siRNA group

**Fig. 7:**
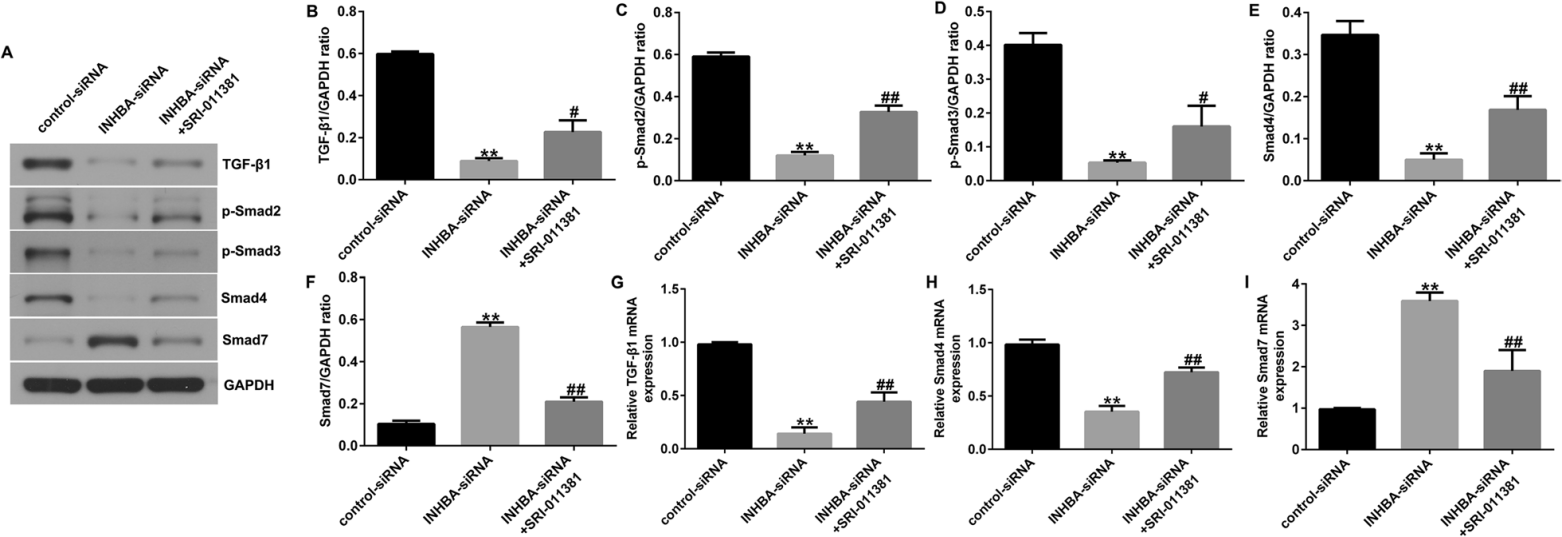
TGF-β1 agonist reversed the effects of INHBA silencing on the TGF-β signaling pathway in MG63 cells. **A**–**I** western blotting and RT-qPCR detected the protein and mRNA expression of genes related to the TGF-β signaling pathway in MG63 cells. **p < 0.01 versus control-siRNA group; #, ##*p* < 0.05, 0.01 versus INHBA-siRNA group

**Fig. 8:**
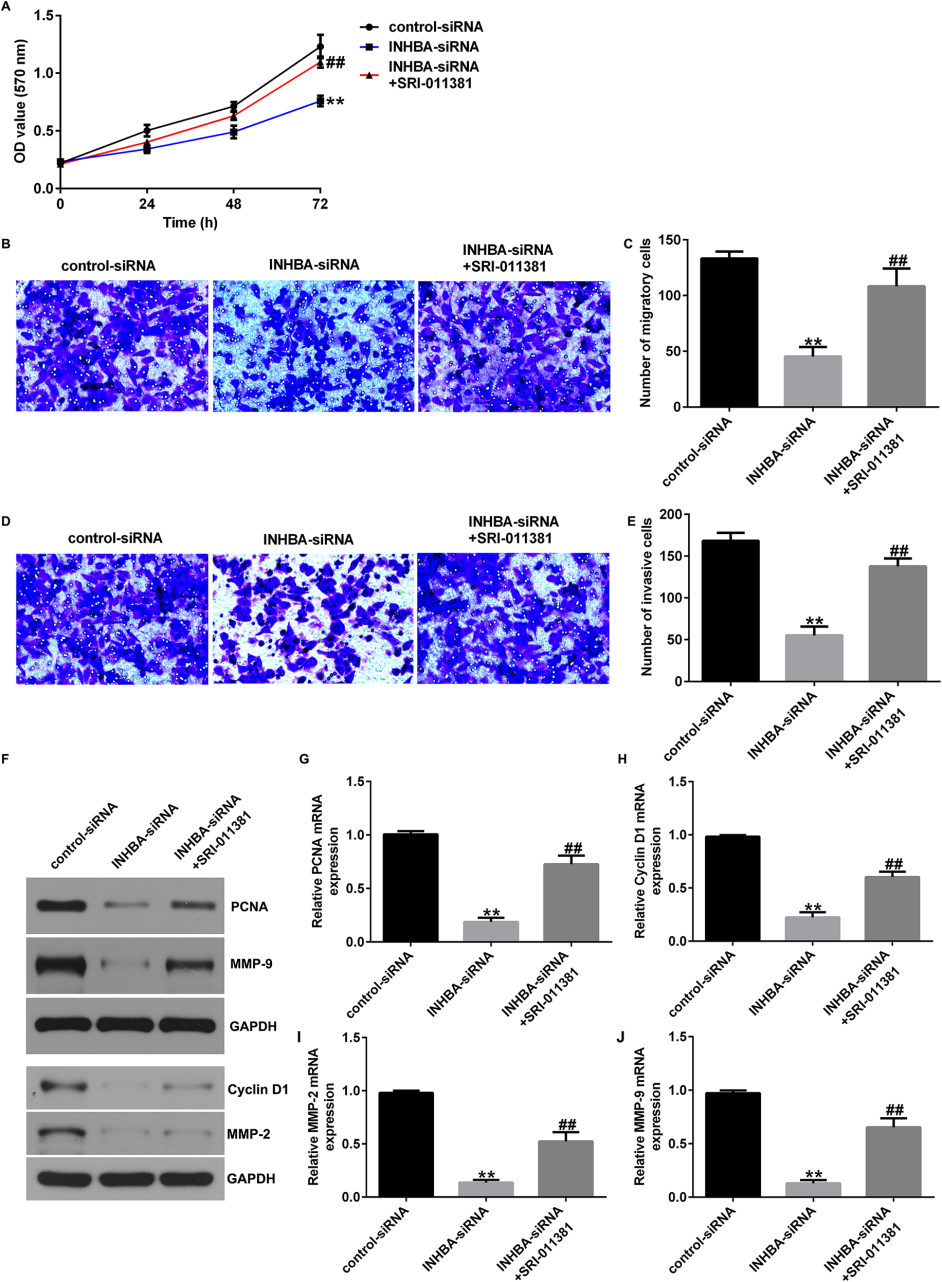
TGF-β1 agonist reversed the effects of INHBA-siRNA on the proliferation, migration, and invasion of U2OS cells. **A** MTT assessment of U2OS cell proliferation; **B**–**E** Transwell assay of U2OS cell migration and invasion; **F**–**J** Analysis of PCNA, Cyclin D1, MMP-2, and MMP9 protein and mRNA expression in U2OS cells by western blotting and RT-qPCR. **p < 0.01 versus control-siRNA group; ##*p* < 0.01 versus INHBA-siRNA group

**Fig. 9:**
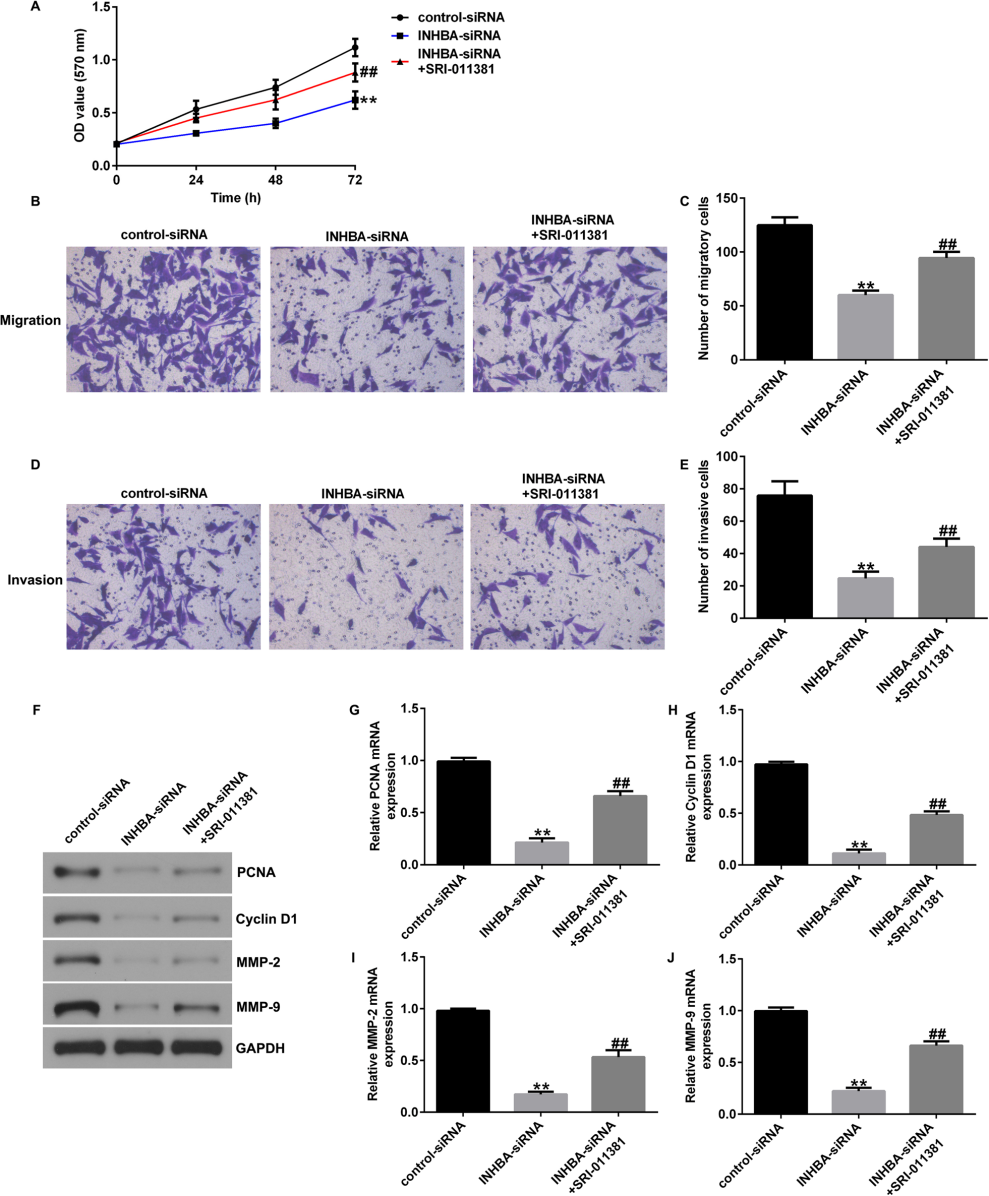
TGF-β1 agonist reversed the effects of INHBA-siRNA on the proliferation, migration, and invasion of MG63 cells. **A** MTT assessment of MG63 cell proliferation; **B**–**E** Transwell assay of MG63 cell migration and invasion; **F**–**J** Analysis of PCNA, Cyclin D1, MMP-2, and MMP9 protein and mRNA expression in MG63 cells by western blotting and RT-qPCR. **p < 0.01 versus control-siRNA group; ##*p* < 0.01 versus INHBA-siRNA group

To further investigate the relationship between the TGF-β signaling pathway and INHBA in OS, we transfected INHBA-siRNA in U2OS or MG63 cells and cultured the cells with TGF-β1 agonist SRI-011381 hydrochloride. After 48 h, the proteins and genes related to the TGF-β signaling pathway were detected with western blotting and RT-qPCR. Silencing of INHBA significantly inhibited the levels of TGF-β1, p-Smad3, p-Smad2, and Smad4 and promoted the levels of Smad7 in U2OS cells (Fig. 6A–F) and in MG63 cells (Fig. 7A–F). However, SRI-011381 treatment significantly increased the levels of TGF-β1, p-Smad3, p-Smad2, and Smad4, and decreased Smad7 in U2OS cells (Fig. 6A–F) and in MG63 cells (Fig. 7A–F). Similarly, INHBA-siRNA significantly inhibited mRNA levels of Smad4 and TGF-β1 and enhanced mRNA levels of Smad7 in U2OS cells (Fig. 6G–I) and in MG63 cells (Fig. 7G–I). However, these changes were significantly reversed by SRI-011381 (Figs. 6G–I, 7G–I). MTT and Transwell assays showed that SRI-011381 significantly increased the proliferation, migration, and invasion of U2OS cells (Fig. 8A–E) and MG63 cells (Fig. 9A–E). In addition, SRI-011381 reversed the downregulation of protein and mRNA levels of PCNA, Cyclin D1, MMP-2, and MMP9 caused by INHBA silencing in U2OS cells (Fig. 8F–J) and MG63 cells (Fig. 9F–J). These results demonstrate that the TGF-β signaling pathway is involved in the effect of INHBA on OS cells.

### Activation of the TGF-β signaling pathway reverses the effect of INHBA knockdown on OS cell EMT

**Fig. 10:**
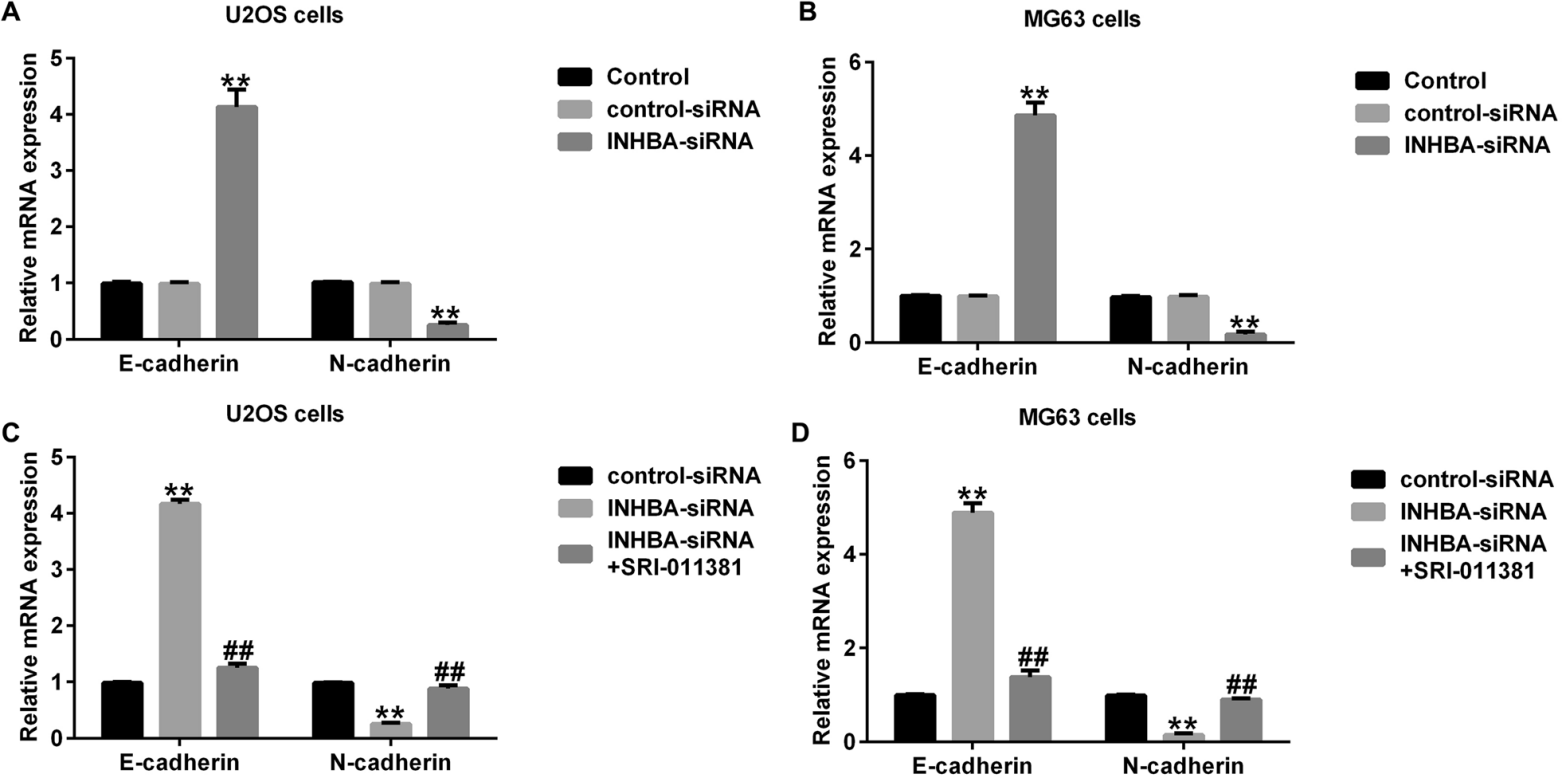
Effect of INHBA-siRNA on OS cell EMT. **A** and **C** E-cadherin and N-cadherin mRNA levels in U2OS cells by RT-qPCR; **B** and **D** E-cadherin and N-cadherin mRNA levels in MG63 cells by RT-qPCR. **p < 0.01 versus control-siRNA group; ##*p* < 0.01 versus INHBA-siRNA group

Finally, we determined the effect of INHBA-siRNA on OS cells EMT. The findings indicated that INHBA-siRNA significantly inhibited OS cell EMT, evidenced by enhanced E-cadherin mRNA expression and reduced N-cadherin mRNA expression in U2OS (Fig. 10A) and MG63 cells (Fig. 10B). As expected, the data of current study also revealed that the effects of INHBA-siRNA on E-cadherin and N-cadherin mRNA in U2OS (Fig. 10C) and MG63 cells (Fig. 10D) were significantly reversed by SRI-011381.

## Discussion

Osteosarcoma is a common primary bone cancer with a high incidence and easy metastasis, and there is currently no effective treatment [1, 2]. Previous studies have found that TGF-β is highly expressed in the serum of patients with OS and promotes OS development [31]. In addition, the TGF-β inhibitor SD-208 significantly reduces the incidence of lung metastases in OS [32]. These results reveal that TGF-β signaling plays an important role in OS development. INHBA, as a member of the TGF-β superfamily, plays an important role in the progression of various cancers through regulating TGF-β signaling pathway [24, 27, 33]. However, the roles and mechanisms underlying OS development have not yet been elucidated.

INHBA is involved in various biological activities, especially tumor development [24]. Recent studies have shown that INHBA expression is elevated in malignant tumors such as gastric, esophageal, and ovarian cancers [19, 23, 34, 35]. INHBA is a prognostic predictor of colon adenocarcinoma [27]. It is upregulated in colon cancer, and its inhibition affects the proliferation, migration, and invasion of colon cancer cells [36, 37]. By activating the TGF-β signaling pathway, INHBA can induce epithelial–mesenchymal transformation (EMT) and accelerate the motility of breast cancer cells [20, 21]. Furthermore, INHBA silencing inhibits the migration and invasion of gastric cancer cells by blocking TGF-β signaling [23, 38]. However, the importance of INHBA in the development of OS has not been systematically studied. We explored the expression of INHBA in patients with OS and OS cell lines and further analyzed the effects of INHBA on the proliferation, migration, and invasion of OS cells.

Samples were collected from 30 patients with OS. The role of INHBA in OS was investigated by analyzing the correlation between INHBA expression in cancerous tissues and pathological parameters, such as tumor stage. The findings of this study showed that the expression of INHBA was enhanced in OS tissues. To investigate the mechanism of action of INHBA in OS development, we examined its function in OS cell lines. The effects and mechanisms underlying INHBA silencing on the proliferation, migration, and invasion of OS cells were investigated using INHBA-siRNA. The results revealed that INHBA levels were enhanced in OS cells and that INHBA silencing significantly inhibited the proliferation, migration, and invasion of OS cells. INHBA has been involved in regulating TGF-β signaling pathway to promote the proliferation, migration, and invasion in cancer cells [23, 26, 34]. In this study, TGF-β signaling pathway was analyzed. As expected, the findings of current study indicated that the silencing of INHBA could inhibit TGF-β pathway in OS cell lines, and TGF-β1 agonist SRI-011381 modified the effects of INHBA silencing on proliferation, migration, and invasion of OS cells.

In conclusion, the downregulation of INHBA inhibits proliferation, migration, and invasion in OS by inhibiting the TGF-β signaling pathway. Furthermore, this study contributes to our understanding of the role of INHBA in OS and identifies new targets for OS treatment.

## Data Availability

The datasets used and/or analyzed during the current study are available from the corresponding author on reasonable request.
