# Correction: INHBA gene silencing inhibits proliferation, migration, and invasion of osteosarcoma cells by repressing TGF‑β signaling pathway activation

**DOI:** 10.1186/s13018-025-05747-7

**Published:** 2025-04-09

**Authors:** Hongyu Zhang, Yuemei Huang, Qiuting Wen, Yan Li, Lin Guo, Na Ge

**Affiliations:** 1 Second Department of Orthopaedics, The Third Affiliated Hospital of Qiqihar Medial University, Qiqihar, 161000 China; 2 Wuzhou Red Cross Hospital, Wuzhou, 543002 China; 3 Department of Clinical Pathology, College of Qiqihar Medical University, Qiqihar, 161006 China; 4 The First Hospital of Qiqihar, Qiqihar, 161005 China; 5 Department of Radiology, The Third Affiliated Hospital of Qiqihar Medial University, No. 27 Taishun Street, Qiqihar, 161000 China

**Correction: Journal of Orthopaedic Surgery and Research (2023) 18:848**

10.1186/s13018-023-04330-2

In this article, Fig. 8 appeared incorrectly and has now been corrected in the original publication. For completeness and transparency, the incorrect and correct versions of Fig. 8 have displayed below.

Incorrect Fig. 8

**Fig. 8:**
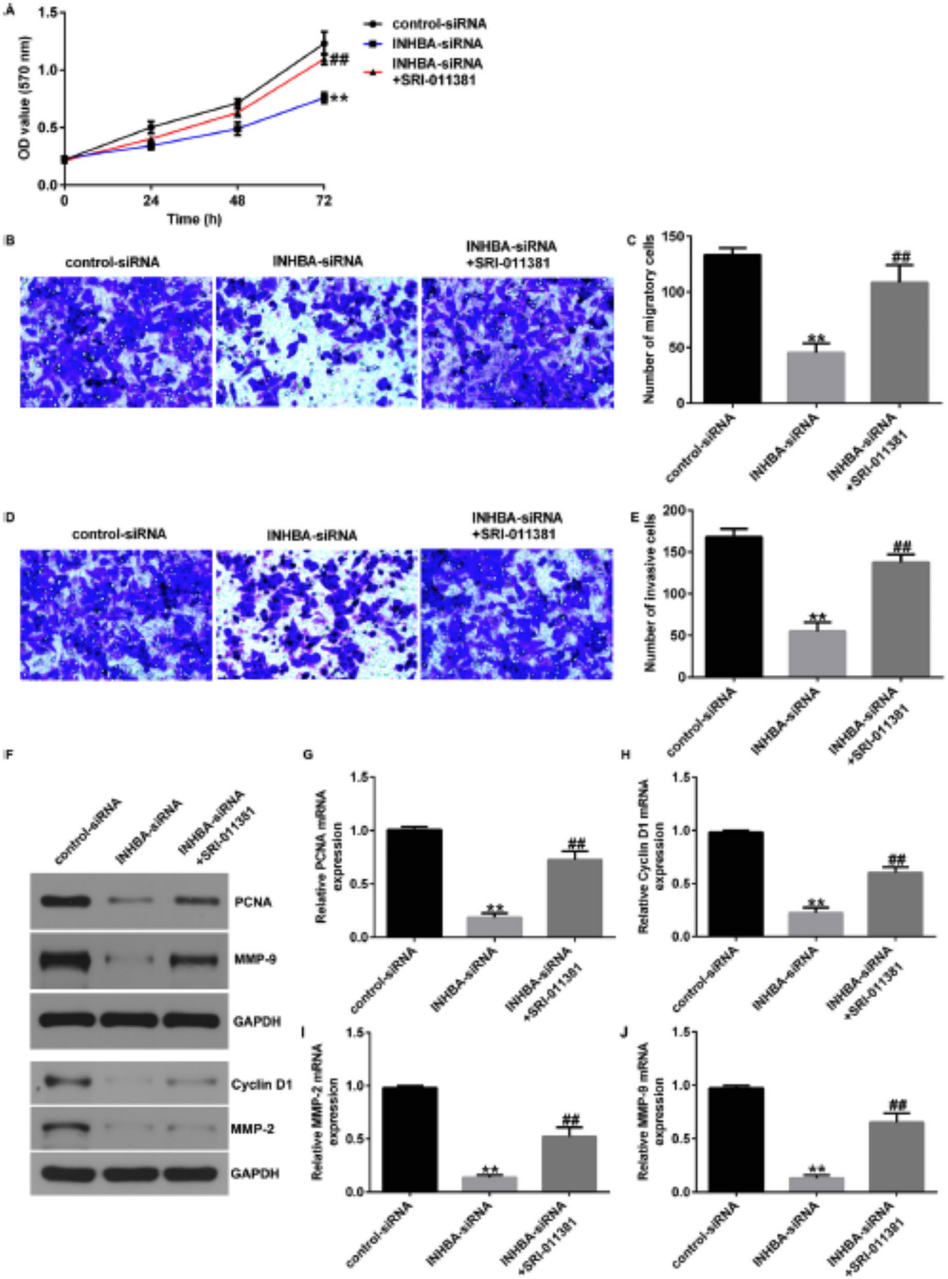
TGF-β1 agonist reversed the efects of INHBA-siRNA on the proliferation, migration, and invasion of U2OS cells. **A** MTT assessment of U2OS cell proliferation; **B**–**E** Transwell assay of U2OS cell migration and invasion; F–J Analysis of PCNA, Cyclin D1, MMP-2, and MMP9 protein and mRNA expression in U2OS cells by western blotting and RT-qPCR. *p<0.01 versus control-siRNA group; ##p<0.01 versus INHBA-siRNA group

Corrected Fig. 8.

**Fig. 8:**
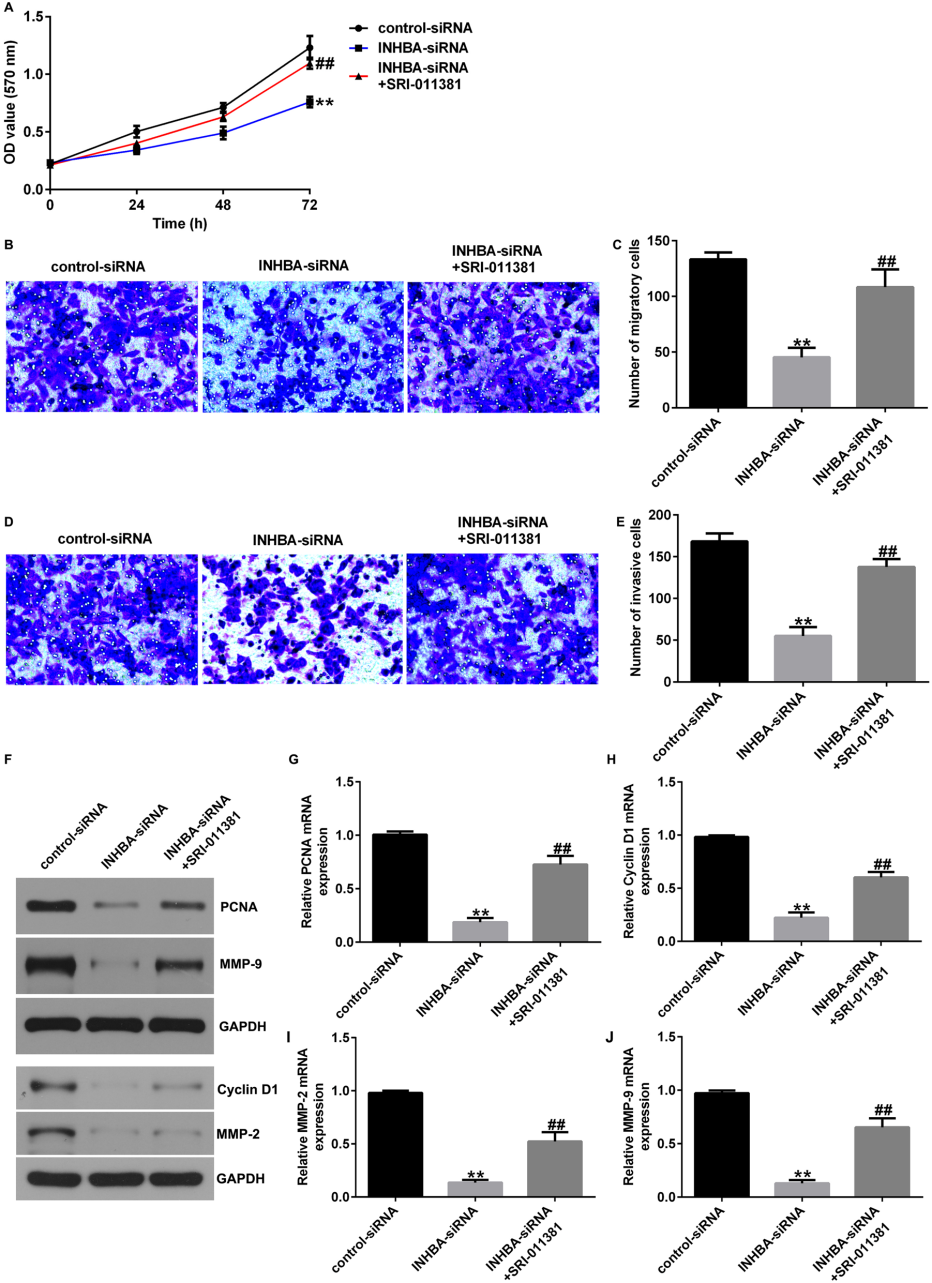
TGF-β1 agonist reversed the efects of INHBA-siRNA on the proliferation, migration, and invasion of U2OS cells. **A** MTT assessment of U2OS cell proliferation; **B**–**E** Transwell assay of U2OS cell migration and invasion; F–J Analysis of PCNA, Cyclin D1, MMP-2, and MMP9 protein and mRNA expression in U2OS cells by western blotting and RT-qPCR. *p<0.01 versus control-siRNA group; ##p<0.01 versus INHBA-siRNA

The original article has been corrected.

